# Shortcuts to Adiabaticity for Optical Beam Propagation in Nonlinear Gradient Refractive-Index Media

**DOI:** 10.3390/e22060673

**Published:** 2020-06-17

**Authors:** Qian Kong, Huimin Ying, Xi Chen

**Affiliations:** 1 International Center of Quantum Artificial Intelligence for Science and Technology (QuArtist) and Department of Physics, Shanghai University, Shanghai 200444, China; kongqian@shu.edu.cn (Q.K.); yinghuimin@shu.edu.cn (H.Y.); 2 Department of Physical Chemistry, University of the Basque Country UPV/EHU, Apartado 644, 48080 Bilbao, Spain

**Keywords:** shortcuts to adiabaticity, non-adiabatic compression, nonlinear beam propagation

## Abstract

In recent years, the concept of “shortcuts to adiabaticity" has been originally proposed to speed up sufficiently slow adiabatic process in various quantum systems without final excitation. Based on the analogy between classical optics and quantum mechanics, we present a study on fast non-adiabatic compression of optical beam propagation in nonlinear gradient refractive-index media by using shortcuts to adiabaticity. We first apply the variational approximation method in nonlinear optics to derive the auxiliary equation for connecting the beam width with the refractive index of the medium. Then, the gradient refractive index is inversely designed through the perfect compression of beam width with the appropriate boundary conditions. Finally, the comparison with conventional adiabatic compression is made, showing the advantage of our shortcuts.

## 1. Introduction

In recent decades, beam propagation in the paraxial approximation has been extensively investigated for many years in the applications of integrated optics waveguide and optical communications [1,2,3]. With the advent of all-optical networks, the novel directional couplers, polarizers, optical dense wavelength division multiplexing (DWDM) are of crucial importance in a photonic integrated circuit, which is a device that integrates multiple (at least two) photonic functions and such is similar to an electronic integrated circuit [4]. Among them, quantum-classical analogies give rise to the elegant ideas for developing above-mentioned optical elements in terms of the similarity between the Helmholtz equation and the Schrödinger equation [5,6]. In detail, adiabatic passages and their variants, including rapid adiabatic passage (RAP) and stimulated Raman adiabatic passage (STIRAP) in quantum optics, provide several approaches to realize optical waveguide couplers and beam splitters with high efficiency and good tolerance [7,8,9]. More recently, two concepts, the adiabatic elimination and STIRAP, have been combined to achieve the all-optical coherence control of optical transmission in three-coupled waveguides, with emphasis on the applications to the state-of-the-art integrated optics and optical quantum computing [10]. Moreover, other interesting cases have been studied in optical waveguides with nonlinearity [11] and PT-symmetry [12], where the adiabatic criteria could be influenced or improved. However, the length of most optical devices designed from adiabatic passages should be long enough to fulfill adiabatic criteria, which does not meet the requirement of miniature in integrated optics.

In recent years, the techniques of “shortcuts to adiabaticity" (STA) have been put forward to reproduce the slow adiabatic but within short time, see reviews [13,14]. It includes two common protocols, invariant-based inverse engineering [15,16] and counter-diabatic driving (or equivalently transitionless quantum algorithm) [17,18,19,20]. In particular, the inverse engineering based on Lewis–Riesenfeld invariant [21] has been first proposed in Ref. [16] for frictionless atom cooling in harmonic traps. In this manner, the trap frequency is inversely designed from the Ermakov equation derived from the dynamical invariant, such that the fast expansion of harmonic trap is achieved without final excitation or heating. Immediately afterwards, such theoretical proposals have been experimentally verified for the frictionless cooling of cold atoms and Bose-Einstein condensates in magnetic traps [22,23]. Moreover, this has been applicable to other systems which most closely resemble the harmonic oscillator and two or three-level systems [24], i.e., mechanical resonator [25], classical RCL circuit [26], photonic lattice [27,28], nitrogen-vacancy (NV) center spin [29,30], and superconducting circuit [31]. More interestingly, there exists freedom left in the method of inverse engineering by only fixing the boundary condition, thus the optimal control theory can be supplemented with perturbation theory to maximize the stability of shortcuts-to-adiabaticity protocols with respect to noise and systematic errors [32,33,34]. In this context, the compatible coupled optical waveguides have been designed from STA, and the better tolerances with respect to input wavelength and spacing errors are achieved as well [35,36,37,38,39]. Similar ideas can be applicable to mode converters [40], polarization rotators [41] and waveguide junctions [42] in the applications of optical waveguides.

The variational approximation method is commonly used for analyzing the stability and collapse of soliton solution in nonlinear optics [43,44] and Bose-Einstein condensates [45,46]. Very recently, we have managed to combine variational approximation and inverse engineering for designing the STA in the more complex systems, where there does not exist dynamical invariant and invariant-based inverse engineering cannot be directly applied. For instance, the fast non-adiabatic soliton compression [47] and consequent nonlinear Feshbach heat engine [48] have been studied by using variational control implementing STA. Lately, these results have been also extended to the shortcuts-to-adiabaticity controls for temporal soliton in optical waveguide [49], coherent many-body particles in power-law potentials [50] and transmon superconducting qubits [51].

In this article, we combine variational approximation and inverse engineering to design fast optical beam propagation in nonlinear gradient refractive-index media. In contrast to moment analysis of paraxial propagation [52], we first apply variational approximation to derive the auxiliary differential equation for connecting the beam width with the gradient refractive index. Then, we design STA protocols for fast compression by engineering gradient refractive index, as compared to the previous adiabatic pulse compression/decompression in optical fiber [53,54]. These results can be also exploited in the optical fiber with the nonlinear effect induced from absorbing atoms [55].

The paper is organized as follows. In Section 2, the variational approximation method is applied to find the auxiliary differential equation to connection the beam width with the guiding coefficient, which is basic for implementing STA. After determining the adiabatic beam propagation as a reference in Section 3, we elaborate the inverse engineering for speed up, by choosing appropriate boundary conditions in Section 4. The numerical simulations are also performed through the beam propagation method for checking the stability. Finally, brief conclusion is presented in Section 5.

## 2. Variational Approximation Method

We consider the one-dimensional optical beam propagation along the *z* axis in a local medium with a longitudinal modulation of the refractive index. In this case, the evolution of the monochromatic optical beam in paraxial approximation is governed by the following (dimensionless) nonlinear Schrödinger equation (NLS) [52]:

(1) $$i\frac{\partial u}{\partial z}+\frac{1}{2}\frac{\partial^{2}u}{\partial x^{2}}+{\left|u\right|}^{2}u-\alpha^{2}\left(z\right)x^{2}u=0.$$

Here *z* is the propagation coordinate, normalized by the diffraction length $z_{R}=2\pi w_{0}^{2}/\lambda$, and *x* is the transversal coordinate, normalized by the beam waist $w_{0}$ [56,57], where λ is the wavelength (the vacuum wavelength $\lambda_{0}$ divided by *n*, the index of refraction) and $w_{0}$ is the beam waist, the radial size of the beam at its narrowest point. *u* is the slowly varying envelope of the spatial part of the electric field, α(z) is guiding coefficient, related to the refractive index of the medium and the wave vector, and the Kerr-type nonlinear coefficient is constant. Noting that we do not consider the gain and loss in the optical fiber, but Equation (1) is still valid, with modifying the effective coefficient of Kerr-type nonlinearity [54]. In such nonlinear system, the dynamics can be described approximately with the following Gaussian ansatz

(2) $$u\left(x,z\right)=A\left(z\right)e^{-\frac{x^{2}}{2a^{2}\left(z\right)}}e^{i\theta \left(z\right)+ic\left(z\right)x^{2}},$$

where *u* is the optical wave field with A(z), a(z), θ(z) and c(z) being the complex amplitude, width, phase, and curvature of the beam respectively. According to the normalization, ${\int |u|}^{2}dx=P$, the input power *P* fulfills $P=aA^{2}\sqrt{\pi }$.

Using the variational approximation method, the re-normalized Lagrangian density corresponding to Equation (1) is given in the following form:

(3) $$L=\frac{i}{2}\left(u^{*}\frac{\partial u}{\partial z}-u\frac{\partial u^{*}}{\partial z}\right)-\frac{1}{2}\frac{{\left|\partial u\right|}^{2}}{\partial x}+\frac{1}{2}{\left|u\right|}^{4}-\alpha^{2}\left(z\right)x^{2}{\left|u\right|}^{2}.$$

Substituting Equation (2) into the above Lagrangian density Equation (3) and integrating over *x*, we obtain the averaged Lagrangian as

(4) $$\begin{array}{c} L=\int Ldx=-A^{2}a\sqrt{\pi }\frac{d\theta }{dz}-\frac{A^{2}a^{3}\sqrt{\pi }}{2}\frac{dc}{dz}-\frac{A^{2}\sqrt{\pi }}{4a}-A^{2}\sqrt{\pi }c^{2}a^{3}+\frac{A^{4}\sqrt{\pi }a}{2\sqrt{2}}-\frac{A^{4}\sqrt{\pi }\alpha^{2}a^{3}}{2}. \end{array}$$

According to the Euler-Lagrange equations,

(5) $$\frac{d}{dz}\left(\frac{\partial L}{\partial {q_{j}}^{\prime }}\right)-\frac{\partial L}{\partial q_{j}}=0,\;\;\;q_{j}\equiv \left\{a,c,\theta \right\},$$

we follow the standard procedures of the variational approach, and finally obtain

(6) $$\begin{array}{c} \frac{\delta L}{\delta a}=-Pa\frac{dc}{dz}+\frac{P}{2a^{3}}-\frac{P^{2}}{2\sqrt{2\pi }a^{2}}-2Pc^{2}a-P\alpha^{2}a=0, \end{array}$$

(7) $$\begin{array}{c} \frac{\delta L}{\delta c}=-2Pca^{2}+Pa\frac{da}{dz}=0, \end{array}$$

(8) $$\begin{array}{c} \frac{\delta L}{\delta \theta }=0. \end{array}$$

It should be noted that Equation (8) implies that θ does not play any role in the variational dynamics. Moreover, from Equations (6) and (7), we obtain the auxiliary differential equation

(9) $$\frac{d^{2}a}{dz^{2}}+2\alpha^{2}\left(z\right)a=\frac{1}{a^{3}}-\frac{P}{\sqrt{2\pi }}\frac{1}{a^{2}},$$

which connects the dynamic evolution of the beam width with the guiding coefficient α(z). This resembles the generalized Ermakov equation obtained for effectively one-dimensional Bose-Einstein condensates (BECs) governed by the Gross–Pitaevskii equation including the nonlinear atom-atom interaction and the time-varying harmonic trap [58]. As a result, the width of the optical beam *a* can be manipulated by modulating the parabolic profile of refraction index through Equation (9), in the presence of Kerr-type nonlinearity. Normally, one can solve Equation (9) numerically to achieve the beam propagation, when the profile of refractive index is fixed. However, the propagation distance should be long enough to fulfill the adiabatic criteria, thus avoiding the distortion (or transition). Now, sharing the concept of STA in Refs. [16,47,58], our idea presented here is to first choose the trajectory of *a* by fixing the initial and final boundary conditions, and later the profile of refractive index is designed inversely, such that one can always implement the same task but within a shorter propagation distance.

## 3. Effective Potential and Adiabatic Reference

The variational method for STA is applied in particular systems that cannot be treated by means of other existing approaches (invariant-based inverse engineering and counter-diabatic driving). Our main motivation is to design STA for fast compression of optical beam from initial profile u(x,0) to final one $u(x,z_{f})$ by designing the parabolic refractive index. After obtaining Equation (9), we shall analyze the stability of beam propagation in terms of perturbative Kepler problem [59], to determine the adiabatic reference for further acceleration.

We assume that Equation (9) is analogous to the classical Newton equation for a fictitious particle with unit mass moving in a parabolic potential with the perturbation resulting from the nonlinear interaction. The classical one-particle system is conservative, and the total energy *E* reads

(10) $$E={\dot{a}}^{2}/2+V\left(a\right),$$

where the first term is kinetic energy (The dot refers to derivative with respect to *z*) and the second term is the potential energy V(a) is

(11) $$V\left(a\right)=\frac{1}{2a^{2}}-\frac{P}{a\sqrt{2\pi }}+\alpha^{2}\left(z\right)a^{2}.$$

**Figure 1:**
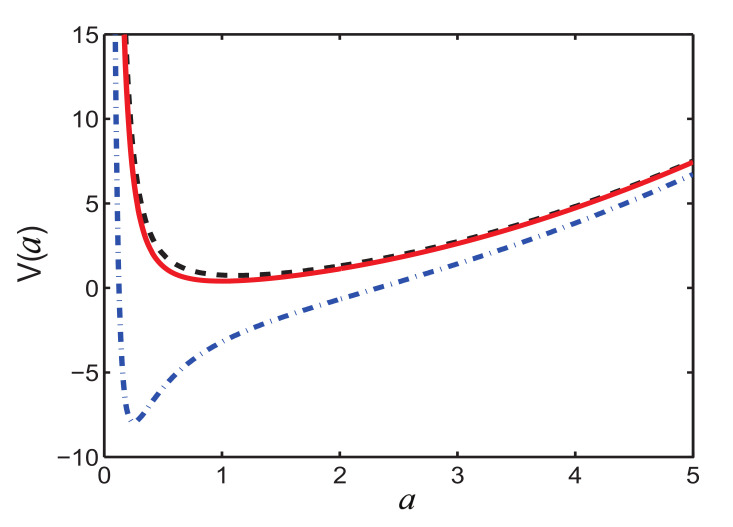
The potential energy V(a) for a fictitious particle with unit mass as the function of the beam width *a*. Here the parameters are $\alpha^{2}\left(0\right)=0.3$ and P=1 (solid red), P=0.1 (dashed black), and P=10 (dash-dotted blue).

From Figure 1, we can see that V(a) has a minimum point, corresponding to the stationary propagation of optical beam, since the total energy (potential energy) for the fictitious particle is minimized with zero kinetic energy. Moreover, according to Figure 1, the optical beam is supposed to be more localized with increasing *P*, as the equilibrium width *a* at the minimum of V(a) becomes smaller. This makes our variational approximation invalid at certain point. Classical analogy implies that the stable equilibrium exists at the minimum of the potential energy. The mass center of optical beam determined by the width *a* oscillates back and forth around this point due to the force −∂V/∂a. Aiming to obtain the stationary solution as an adiabatic reference, we choose

(12) $$\alpha^{2}\left(z\right)=Ae^{\beta z}.$$

According to ∂V/∂a=0, the adiabatic reference can be achieved as the solution of

(13) $$\frac{1}{a_{c}^{3}}-\frac{P}{\sqrt{2\pi }}\frac{1}{a_{c}^{2}}-2\alpha^{2}\left(z\right)a_{c}=0,$$

which yields the initial and final beam widths,

(14) $$a_{c}\left(0\right)=1,\;\;a_{c}\left(z_{f}\right)=0.5,$$

with the parameters A=0.3, β=0.01, P=1, $z_{f}=306$. In this case, it implies that ${\ddot{a}}_{c}\left(0\right)={\ddot{a}}_{c}\left(z_{f}\right)=0$ naturally, see the detailed discussions later on the boundary conditions. Therefore, we can use the long propagation distance $z_{f}=306$ with such gradient refractive index to compress the width of optical beam by half. In what follows we will use STA protocol to shorten the propagation distance, while keeping the same result.

## 4. Inverse Engineering for Fast Compression

Now it is ready for us to design STA based on the inverse engineering [47,58] to compress the optical beam within finite short propagation distance $z_{f}$. To this end, we design a(z) by assuming the following simple polynomial ansatz [16,47,58]

(15) $$a\left(z\right)=a_{i}-6\left(a_{i}-a_{f}\right)s^{5}+15\left(a_{i}-a_{f}\right)s^{4}-10\left(a_{i}-a_{f}\right)s^{3},$$

with $s=z/z_{f}$, satisfying the boundary conditions

(16) $$\begin{array}{c} a\left(0\right)=a_{i},\;\;a\left(z_{f}\right)=a_{f}, \end{array}$$

(17) $$\begin{array}{c} \dot{a}\left(0\right)=\dot{a}\left(z_{f}\right)=\ddot{a}\left(0\right)=\ddot{a}\left(z_{f}\right)=0. \end{array}$$

Here the boundary conditions (16) should coincide with the initial and final values of beam width (14), given by the adiabatic reference, such that $a_{i}=a_{c}\left(0\right)$ and $a_{f}=a_{c}\left(z_{f}\right)$, and the other boundary conditions are postulated for the smooth changes of refractive index at initial and final edges. Interestingly, these boundary conditions (17) also imply the minimum potential energy V(a) and zero kinetic energy at initial and final points, since the first and second derivatives of *a* are null. This guarantees that our designed STA protocol can compress the optical beam with the same initial and final conditions as the adiabatic reference. Of course, different ansatz can be also used for interpolating the six boundary conditions mentioned above. However, they are not optimal at all. One can further optimize it, for instance, by minimizing the propagation distance [58].

**Figure 2:**
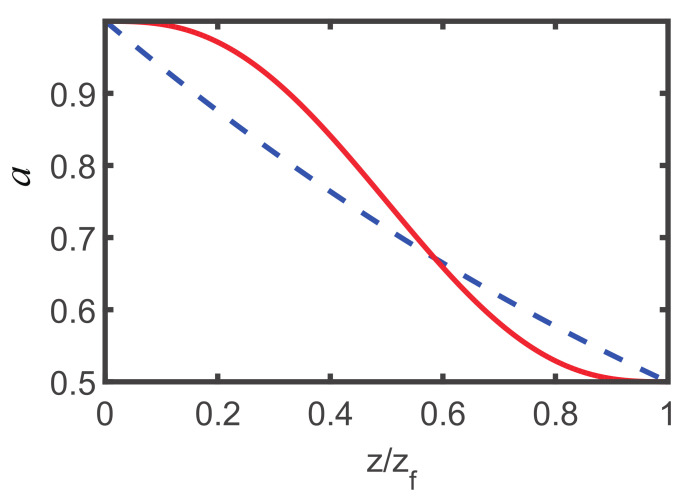
The evolution of beam width designed by STA (solid red) and adiabatic protocols (dashed blue), where the initial and final values are fixed by Equation (14). Parameters: $a\left(0\right)=a_{i}=1$ and $a(z_{f})=0.5$ are the same for STA and adiabatic protocols, but the propagation distance $z_{f}=306$ (adiabatic) and $z_{f}=5$ (STA) are different. P=1 and the other parameters are the same as those in Figure 1.

Figure 2 depicts the evolution of beam width designed by STA (solid red) and adiabatic protocols (dashed blue), where the propagation distances $z_{f}=306$ (adiabatic) and $z_{f}=5$ (STA) are different. By using polynomial ansatz (15), we interpolate the STA evolution of beam width by fixing the initial and final boundary conditions (14) solved from an adiabatic reference. Obviously, the trajectory a(z) designed from STA does not follow the adiabatic one $a_{c}\left(z\right)$, the solution of Equation (13). From Figure 2, it is obvious that the beam width can be compressed from its initial value a=1 to final value a=0.5 after propagating a distance $z_{f}=306$ for adiabatic case while it could be achieved only after propagating a distance z=5 in STA case. In the experiment [56], the relevant parameters are $\lambda_{0}=0.5\;\mu$m, n=2.3, and the divergence of Gaussian beam, $\theta_{0}=15.7$ mrad, such that the diffraction length distance $z_{R}=2\pi w_{0}^{2}n/\lambda_{0}≃561.465\;\mu$m, determined by the beam waist $w_{0}=\lambda_{0}/\pi n\theta_{0}≃4.4075\;\mu$m. Therefore, the propagation distance can be significantly reduced from $z_{f}=17.18$ cm to $z_{f}=0.28$ cm in nonlinear media.

**Figure 3:**
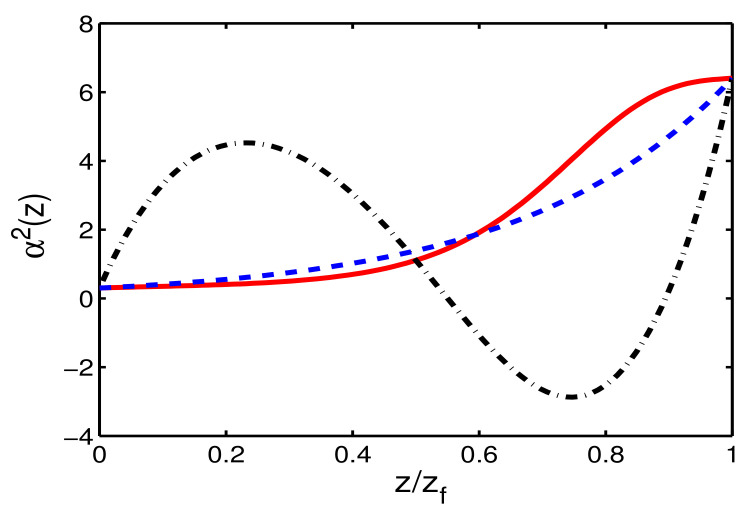
Guiding coefficient, $\alpha^{2}\left(z\right)$, relevant to the parabolic profile of refractive index, for designed STA and adiabatic protocols, when $z_{f}=5$ (solid red), $z_{f}=0.6$ (dot-dashed black) for STA protocols are compared with adiabatic references $z_{f}=306$ (dashed blue).

Next, we can engineer the guiding coefficient, relevant to the parabolic profile of refractive index, through the generalized Ermakov equation, see Equation (9), once the trajectory a(z) is fixed. In Figure 3, we compare the function of $\alpha^{2}\left(z\right)$, designed from STA and adiabatic protocols with different distances $z_{f}$. Their initial and final values $\alpha^{2}\left(0\right)=0.03$ and $\alpha^{2}\left(z_{f}\right)=6.4$ are determined from Equation (13), which are consistent for both STA and adiabatic protocols due to the boundary conditions. However, there are two different things required for further clarification. The profile of refractive index for adiabatic reference is exponentially growing function (12), which is not smooth at z=0 and $z=z_{f}$. Moreover, the profile of refractive index for STA is not as smooth as the adiabatic one, it will be changed from attractor to repeller when the propagation distance becomes shorter. This could set the physical constraints on STA, when the refractive index requires to be a negative value, leading to the loss of optical beam in practical metamaterial.

**Figure 4:**
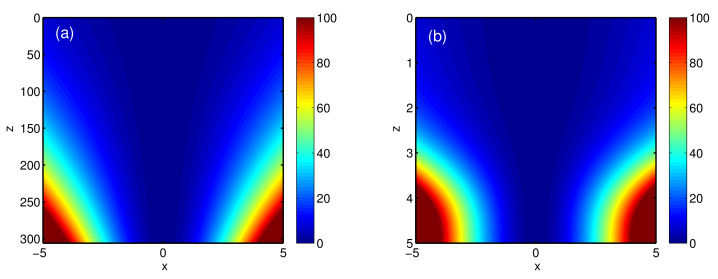
Density plot of effective potential, $\alpha^{2}\left(z\right)x^{2}$, corresponding to the parabolic profile of the refractive index, where the adiabatic reference (**a**) and STA protocol (**b**) are presented with the same parameters as those in Figure 2.

To give the intuitive picture, the density plot of the effective potential, $\alpha^{2}\left(z\right)x^{2}$, i.e., the parabolic profile of refractive index, is further shown in Figure 4. We can clearly see that the parabolic profile of refractive index changes drastically for STA protocol, while the one for adiabatic reference is smooth. In addition, this tell us the effective potential is similar to the time-modulating harmonic traps for atom cooling, which can be implemented by the refractive-index gradient along the radial direction, for instance, in the core of a graded-index optical fiber [52]. Since the designed refractive index is $n\propto \alpha^{2}\left(z\right)x^{2}$, the effective potential becomes repulsive repeller for a very short distance, which implies the negative refractive index. In this situation, the experimental implementation could be more complicated, and the loss in metamaterial with negative refractive index also sets the limit to STA in such optical systems.

**Figure 5:**
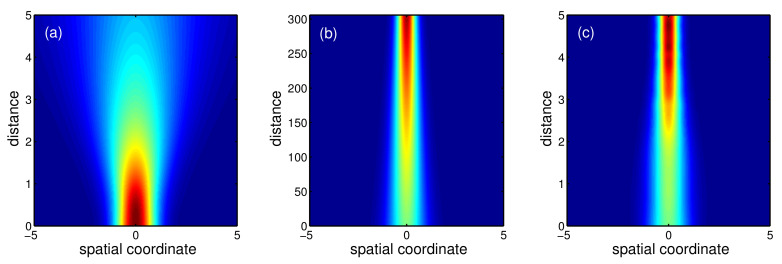
Beam propagation by using the split-step Fourier method, for free space without parabolic refractive index (**a**), adiabatic reference (**b**), and STA protocol (**c**). All the parameters are the same as those in Figure 2.

By using a pseudo-spectral numerical method [4], i.e., split-step Fourier method (or beam propagation method), we solve NLS (1) with our designed guiding coefficients. Figure 5a shows that in a Kerr-type nonlinear medium, the Gaussian beam is diffracted during transmission without the parabolic refractive index, and the beam width becomes boarder. This clarifies the significant role of parabolic refractive index. Figure 5b,c further illustrate the beam propagation in the presence of different parabolic refractive index, corresponding to Figure 4a,b. The numerical simulation confirms the beam width of optical beam can be compressed from a((0)=1 to $a(z_{f})=0.5$ by using adiabatic and STA protocols. However, there is remarkable difference between the resulting propagation distances. From Figure 5, the adiabatic propagation distance requires $z_{f}=306$, while the STA one is decreased up to $z_{f}=5$. All results are consistent with the theoretical predictions. By inversely engineering the refractive index, one can compress the optical beam efficiently within a short propagation distance.

**Figure 6:**
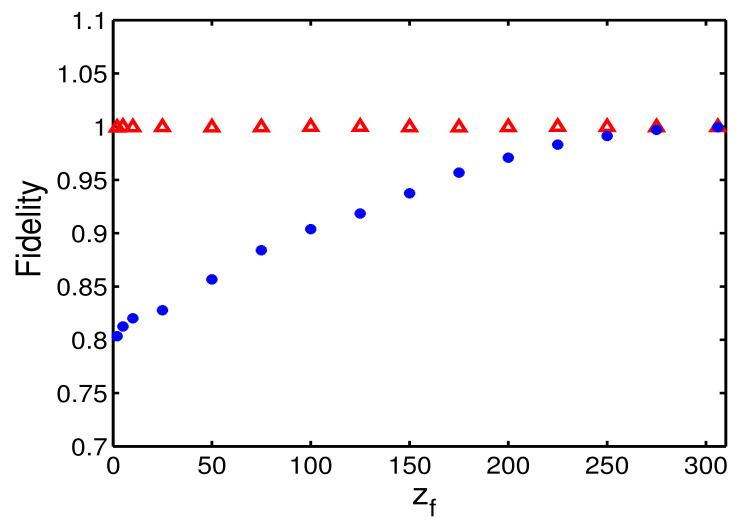
Fidelity versus the propagation distance $z_{f}$ for adiabatic reference (blue dotted) and STA protocol (red dotted). All parameters used here are the same as those in Figure 2.

Finally, we shall discuss the validity of our method. We check the fidelity for different methods as the function of propagation distance $z_{f}$, in Figure 6, where the fidelity is defined as

(18) $$F=|\langle u^{ref}\left(x,z_{f}\right)|u\left(x,z_{f}\right)\rangle {|}^{2},$$

with $u^{ref}\left(x,z_{f}\right)$ being the targeted solution, see Equation (2), and $u(x,z_{f})$ being the final numerical result. In Figure 6, we find that the fidelity of STA protocol is almost 1, while it decreases dramatically for adiabatic reference. Clearly, the adiabatic compression by using the guiding coefficient (12) requires the propagation distance longer than $z_{f}=250$ while the propagation distance can be decreased up to $z_{f}=5$. Moreover, one can try the bright-soliton ansatz of hyperbolic secant. However, when the Kerr-type nonlinearity is weak, the Gaussian assumption is still reasonable of soliton, see the similar analysis for Bose-Einstein condensates in Refs. [45,47,58]

## 5. Conclusions

In summary, we have proposed an efficient method for fast non-adiabatic compression or decompression of optical beam propagation in nonlinear gradient refractive-index media. This is the optical analogy of frictionless expansion/compression of weakly interacting atoms in time-modulating harmonic traps [47,58]. We apply the variational approximation method to NSL equation, describing the monochromatic beam propagation in paraxial approximation. Consequently, the auxiliary differential equation is obtained to connect the beam width with the guiding coefficient. Then, the parabolic refractive index is designed inversely by fixing the boundary conditions, determined by an adiabatic reference. By using split-step Fourier method, we confirm numerically that STA manages to compress the optical beam with much shorter propagation distance, as compared to adiabatic reference.

Finally, we should mention several extensions for further exploration. For example, one can combine the optimal control theory and inverse engineering for minimize the distance [58]. Instead of parabolic refractive index, the Kerr-type nonlinearity can be also engineered for designing STA. Moreover, the gain/loss [53,54], cubic/quintic nonlinearity, distributed dispersion [60] and even absorbing atomic media [55] in such optical systems provide alternatives with more flexibility. Our method proposed here is of interest with other applications of pulse compression in nonlinear optical fiber, waveguide interconnection, mode transformer for miniaturized optical circuits, and others [61,62].
